# Downregulation of Diacylglycerol kinase zeta (DGKZ) suppresses tumorigenesis and progression of cervical cancer by facilitating cell apoptosis and cell cycle arrest

**DOI:** 10.1080/21655979.2021.1918505

**Published:** 2021-04-29

**Authors:** Keying Liu, Biyun Xue, Guiqin Bai, Wentao Zhang

**Affiliations:** a Department of Gynecology and Obstetrics, The First Affiliated Hospital of Xi’an Jiaotong University, Xi’an, Shaanxi Province, China; b Department of Gynecology and Obstetrics, Xi’an North Hospital, Xi’an, Shaanxi Province, China; c Department of Preventive Veterinary Medicine, College of Veterinary Medicine, Northwest A&F University, Yangling, Shaanxi Province, China

**Keywords:** Cervical cancer, diacylglycerol kinase zeta, cell cycle, apoptosis, tumor growth

## Abstract

Diacylglycerol kinase zeta (DGKZ) participates in cancer progression. Here, the current work aims to identify the functional role of DGKZ in cervical cancer (CC). DGKZ expression in cervical cancer tissues and paired adjacent normal cervical tissues was assessed using Immunohistochemistry assay. SiHa and HeLa cells were transfected with lentivirus plasmids (sh-DGKZ or sh-NC) to evaluate the effects of DGKZ knockdown on cell proliferation, apoptosis and cell cycle distribution in vitro. Furthermore, BALB/c nude mice were injected subcutaneously with Lentivirus-sh-DGKZ-SiHa cells or Lentivirus-sh-NC-SiHa cells to analyze the influence of DGKZ silencing on tumor growth of CC in vivo. Moreover, the potential molecular mechanisms were predicted by GO and KEGG analysis and preliminarily explored through PathScan Analysis. Elevated DGKZ expression in cervical tumor was observed. Downregulation of DGKZ repressed proliferation and boosted apoptosis of SiHa and HeLa cells and induced cell cycle arrest at G0/G1 phase. In addition, Knockdown of DGKZ restrained tumor growth in tumor xenograft mice. Importantly, GO and KEGG analysis displayed that differentially expressed proteins induced by silence of DGKZ were mostly enriched in autophagy or mitophagy, indicating that the functions of DGKZ on cell proliferation and tumor growth may be associated with autophagy or mitophagy. PathScan analysis presented that PI3K-AKT and TAK1-NF-κB signaling pathways were prominently inhibited in SiHa cells transfected with sh-DGKZ. In summary, downregulation of DGKZ impeded cell proliferation, boosted cell apoptosis and induced cell cycle arrest to suppress tumorigenesis and progression of cervical cancer.

## Graphical Abstract

**Figure:**
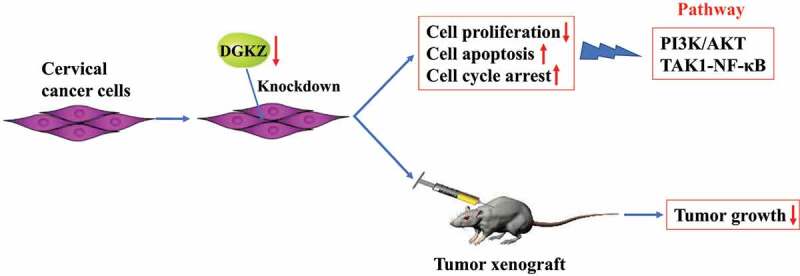

## Introduction

Cervical cancer (CC) is the fourth most deadly cancer and ranked only after breast cancer, colorectal cancer and lung cancer. Detection and prophylaxis of HPV are effective approaches to diagnose and prevent cervical cancer [1]. Surgery, chemotherapy and radiotherapy are the efficient therapies for early stage cervical cancer [2–4]. Nonetheless, the therapeutics are limited due to radioresistance, chemoresistance, and postoperative recurrence, and exploration of the precise molecular mechanism of the pathology and potent targets is of utmost exigency.

Diacylglycerol kinase zeta (DGKZ) belongs to the diacylglycerol kinase (DGK) family which catalyzes diacylglycerol (DAG) into phosphatidic acid (PA) [5]. PA production is the initiation of PtdInsP2 supplement in ‘PtdIns cycle’, which provides a lipid substrate, either for effects of phospholipase C (PLC) hydrolysis or for PtdIns 3 kinase (PI3K) action [5]. DGK, as an immunotherapeutic target in cancer, mainly performs to improve T cell activity [6]. DGK is a second messenger mediating various biological effects. DGKα inhibitors boost apoptosis and reduce viability of cancer cell lines [7]. DGKγ works as a tumor suppressor in hepatocellular carcinoma by negatively regulating glucose transporter 1 [8]. In addition, certain investigations have indicated that DGKZ is an oncogene facilitating cancer progression and development [9,10]. DGKZ activates mammalian target of rapamycin complex 1(mTORC1), thus promoting cancer cell growth and survival [11].

In spite of knowledge mentioned above, the role of DGKZ in CC progression has not been investigated till now. The present work was designed to evaluate the potential of DGKZ in aggravation of CC development. In the current study, we analyzed DGKZ expression in tumor tissues and adjacent normal tissues from CC patients and explored the role of DGKZ in *in vivo* tumor-bearing mouse models and *in vitro* CC cell lines. To further understanding the biological functions of differentially expressed proteins in DGKZ knockdown or normal cervical cancer cells, GO and KEGG pathway analyses were performed. Moreover, PathScan antibody assay was adopted to identify the signaling pathways mediating the effects of DGKZ in CC progression. Findings of current study revealed the effects of DGKZ in accelerating CC progression and could lay theoretical basis for the diagnosis and therapies of CC in clinic.

## Materials and methods

### Cell lines and tissue specimens

Cervical cancer derived cell lines SiHa and HeLa were obtained from the Cell Bank of the Chinese Academy of Sciences (Shanghai, China) and conducted to cell-line authentication using Short Tandem Repeat (STR) profiling by GeneChem (Shanghai, China). All the cells were cultured with Dulbecco’s modified Eagle’s medium (DMEM; Gibco, NY, USA) containing 10% fetal bovine serum (FBS), 100 U/mL penicillin and 100 µg/mL streptomycin at 37°C in 5% CO_2_ atmosphere. 31 pairs of cervical tumors and adjacent tissues of patients who had been diagnosed with cervical cancer and undergone radical resections at Taizhou Hospital of Zhejiang Province were obtained.

### Immunohistochemistry and tissue microarray assay (TMA)

TMA samples of DGKZ were made by Shanghai Outdo Biotech Co. Ltd. Standard indirect immunoperoxidase procedures were used for immunohistochemistry by GeneChem (Shanghai, China). Rabbit polyclonal antibody of DGKZ (sigma-Aldrich) was used to stain on sections from formalin-fixed human cervical tissues.

### Lentivirus-shRNA transfection

The Lenti-shRNA vector system (pGV112) was constructed, packed, purified by GeneChem (Shanghai, China), and manipulated according to the protocol provided by the manufacturer. Then, transfection was performed using Lipofectamine 2000 following the manufacturer’s instructions.

To identify the transfection efficiency, SiHa and HeLa cells were transfected with lentivirus plasmids (sh-DGKZ or sh-NC). 48 h post transfection, DGKZ expression was observed using inverted fluorescence microscope (OLYMPUS, Japan).

### RNA extraction and real-time quantitative PCR (RT-qPCR)

Total RNAs were extracted using SuperfecTRI Total RNA Isolation Reagent (Pufei Biotech Co., Ltd, Shanghai, China). cDNA was synthesized with M-MLV Reverse Transcriptase (Promega, Madison, WI, USA) according to the manufacturer’s instructions. RT-qPCR was performed using SYBR Master Mixture (TAKARA BIO, Japan) following a standard protocol and analyzed using LightCycler480 (Roche, Basel, Switzerland). Glyceraldehyde-3-phosphate dehydrogenase (GAPDH) served as the endogenous control.

### Western blot analysis

Following the designed transfection, SiHa and HeLa cells were lysed in ice-cold NP-40 lysis buffer (Dingguo Biotech, Shanghai, China) and cell lysates were centrifuged at 12,000 rpm for 10 min at 4°C. After that, total protein concentration was determined using BCA method. Equal amount of protein was subjected to SDS-PAGE and then transferred to PVDF membranes (Millipore, MA, USA). Membranes were blocked in 5% skimmed milk, and then incubated with anti-DGKZ antibody (Abcam, ab239080, 1:1000), anti-β-actin antibody (Abcam, ab8227, 1:5000) overnight (4°C). The next day, membranes were incubated with appropriate horseradish peroxidase-conjugated secondary antibodies. The signals of immunoblots were generated by an enhanced chemiluminescence (ECL) kit (Millipore, MA, USA) and visualized using a chemiluminescence system (Bio-Rad, USA). Relative protein level of DGKZ was quantified by densitometry using ImageJ Software and normalized to β-actin.

### Cell proliferation analysis

MTT assay was performed to analyze the proliferation capacity of SiHa and HeLa cells. Briefly, a total of 1 × 10^4^ cells were seeded into 96-well plates. After incubation for 1, 2, 3, 4 and 5 d, 20 µl of MTT (5 mg/mL) was added to each well and incubated at 37°C for 4 h. Next, the medium was changed to 200 µL dimethyl sulfoxide (DMSO). The absorption value was examined at 490 nm on a microplate reader (Bio-Rad, CA, USA). All the experiments were performed in triplicate.

### Cell cycle analysis

Briefly, following the designed transfection, a total of 1 × 10^6^ cells were collected and washed with PBS. Then, cells were fixed in 75% ethanol at 4°C for 2 h. The fixed cells were stained using cell staining reagent (40 × PI (2 mg/mL, sigma-Aldrich): 100× RNase (10 mg/mL, Fermentas): 1 × D-Hanks (pH 7.2) = 25:10:1000) in the dark at room temperature for 20 min. Finally, cells were analyzed by flow cytometry (Guava easyCyte HT, Millipore, MA, USA). The experiment was repeated thrice under the same conditions.

### Apoptotic cell analysis

Transfected cells were washed using prechilled 1 × D-Hanks (pH 7.2) and then 1 × 10^6^ cells were resuspended in 1× binding buffer. 10 μl Annexin V-APC was added into the cells and the mix solution was incubated in the dark at room temperature (25°C) for 15 min. Cell apoptosis analysis was performed by flow cytometry (Guava easyCyte HT, Millipore, MA, USA). The experiment was repeated thrice under the same conditions.

Caspase3/7 activity was detected by Capase glo® 3/7 assay kit (Promega, USA). In short, 100 μl/well cell suspension (1 × 10^4^ cells/well) were pflanted into 96-well plates. The blank control was added 100 μl/well culture medium. Subsequently, 100 μl of Caspase-Glo® 3/7 reagent was added to each well and incubated in the dark at room temperature (25°C) for 1 h. The luminescence was measured on a microplate reader (Bio-Rad, CA, USA).

### Xenograft tumor growth and fluorescence imaging assay

20 BALB/c nude mice were purchased from Shanghai Lingchang systems (Shanghai, China) and housed in a temperature-and humidity-controlled specific pathogen free (SPF) atmosphere (23 ± 2°C, 60–70%). BALB/c mice were subcutaneously injected with Lenti-shRNA DGKZ-SiHa cells or Lenti-shRNA NC-SiHa cells. From the eleventh day of injection, the length and width of xenograft tumors were monitored every 3 days, and the tumor volume was calculated using the formula V = 1/2 × a^2^ × b (‘a’ represents the width and ‘b’ represents the length). At the thirty-first day of injection, animals were anaesthetized with 0.7% pentobarbital and photographed using IVIS Spectrum (Perkin Elmer). Subsequently, weights of xenograft tumors were recorded after sacrificing the mice by injection of 2% pentobarbital and dislocation of cervical vertebra. For determination of xenograft tumorigenesis, the anesthetized BALB/c nude mice were conducted to fluorescence imaging assay using small animal live imaging system. Animal carcasses and body parts were double bagged and transferred to the −20°C refrigerator for the further centralized processing.

### Tandem mass tag (TMT)-based quantitative proteomics and differentially expressed protein analysis

Proteins in Lentivirus-shRNA negative control-SiHa cells and Lentivirus-shDGKZ-SiHa cells were extracted and dissected by trypsin. The peptide fragments were marked using TMT and conducted to quantitative proteomics analysis using Q Exactive (Thermo Fisher Scientific) by GeneChem (Shanghai, China). Using the Human database (Uniprot_HomoSapiens_20386_20180905), MS/MS data were analyzed by Proteome Discoverer 2.1(Thermo Fisher Scientific) with the MASCOT2.6 search engine. Three biological replicates were performed for each sample and three technical replicates (replicate injections) were performed for each biological replicate. The data were filtered according to the standard of false discovery rate (FDR) < 0.01 to obtain highly reliable qualitative results. Differentially Expressed Proteins (DEPs) were analyzed by comparing with biological replications. Two-sided T-tests were performed to evaluate abundance changes of corresponding protein. The one-sample two-sided t-test was a test for difference from 0 on the log2 scale performed with unique peptide ratio of protein. In general, a significance level of 0.05 was used for statistical testing.

### Gene ontology (GO) and kyoto encyclopedia of genes and genomes (KEGG) analysis

Gene Ontology (GO) biological process enrichment of DEPs were annotated by DAVID (https://david.ncifcrf.gov/) [12–14]. Significance enrichment analysis of GO annotation is to evaluate the significance level of protein enrichment in a GO term through Fisher’s Exact Test. Pathways enrichment analysis of DEPs was conducted according to Kyoto Encyclopedia of Genes and Genomes (KEGG). TOP 30 terms were presented. The recommended p-value cutoff of p < 0.05 was used.

### PathScan analysis

PathScan analysis was performed using PathScan® Antibody Array Kit (Cell Signaling Technology, Danvers, MA, USA) following the manufacturer’s protocol [15].

### Statistical analysis

Unless otherwise indicated, all experimental data of at least three independent experiments are presented as mean ± SD with error bars. Statistical analysis was performed using GraphPad Prism version 5.0 (GraphPad Software, San Diego, CA, USA). Differences in indicators between treatment groups and controls were assessed using Student’s t-test. P < 0.05 was considered statistically significant.

## Results

### Elevated expression of DGKZ in CC

**Figure 1.**
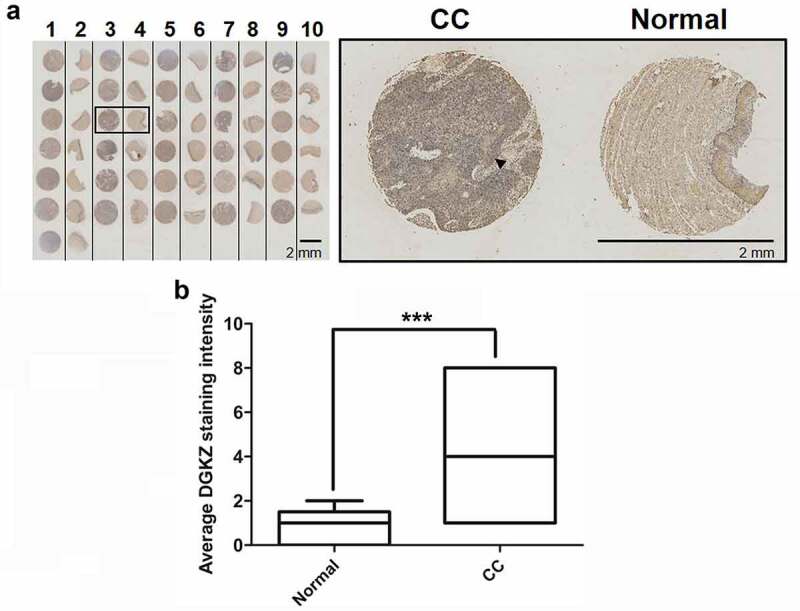
Expression levels of DGKZ in cervical cancer tissues and paired adjacent normal cervical tissues. (a) Immunohistochemical staining and Tissue Microarray Assay (TMA) of DGKZ protein in 31 tumor tissues and 31 adjacent non-tumor tissues from CC patients. (b) Relative expression of DGKZ in CC and normal group quantified by box-and-whisker plot. (***p < 0.001)

For identifying the specific role of DGKZ in CC, DGKZ expression levels in 62 cervical tissues (31 tumor tissues and 31 adjacent non-tumor tissues from CC patients) were assessed using IHC. As it was seen, DGKZ exhibited strong staining in CC tissues with a total positive rate of 90% (23/31), which was significantly higher than 0% (1/31) in representative adjacent normal tissues (Figure 1a). In addition, the distribution of pathological level in normal and CC groups was further quantified using box-and-whisker plot (Figure 1b). The expression level of DGKZ was significantly higher in CC group than that in normal group (4.4 ± 0.09 vs 0.7 ± 0.02, P < 0.001).

*Knockdown of DGKZ suppresses growth of SiHa and HeLa cells and induces cell cycle arrest at G0/G1 phase*

**Figure 2.**
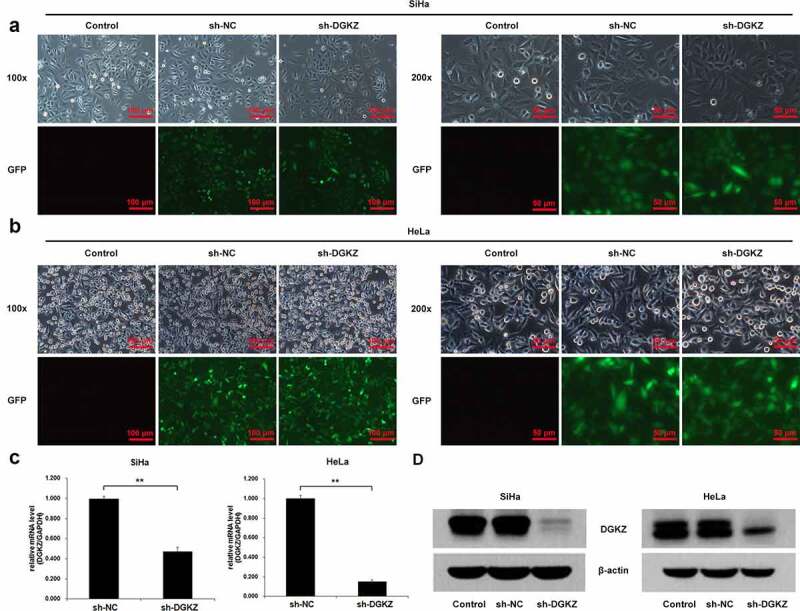
SiHa and HeLa cells were transfected with lentivirus plasmids (sh-NC or sh-DGKZ) to regulate DGKZ expression. (a, b) The efficient silencing of DGKZ expression in SiHa cells and HeLa cells was verified using Immunofluorescence assay. (c)The efficient silencing of DGKZ expression in SiHa and HeLa cells was verified by RT-qPCR. (d) The efficient silencing of DGKZ expression in SiHa and HeLa cells was verified by western blot analysis. (**p < 0.01)

**Figure 3.**
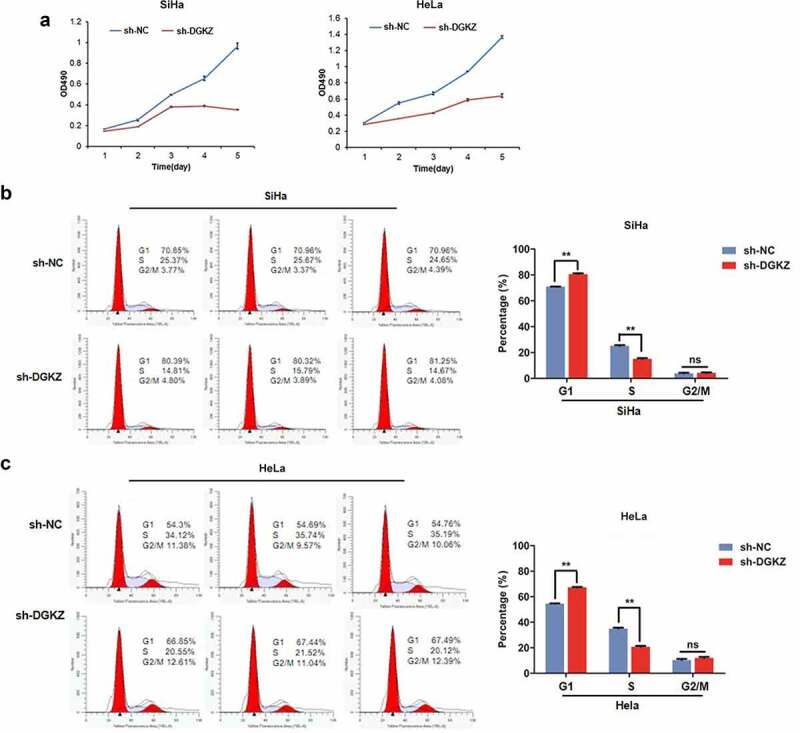
Downregulation of DGKZ repressed proliferation of SiHa and HeLa cells. (a) Proliferation of SiHa and HeLa cells transfected with sh-NC or sh-DGKZ during 5 d was detected by CCK-8 assay. (b, c) Cell cycle distribution of SiHa or HeLa cells transfected with sh-NC or sh-DGKZ was determined by flow cytometry via PI staining. (**p < 0.01)

In order to determine the biology function of DGKZ in CC, SiHa and HeLa cells were transfected with lentivirus plasmids (sh-DGKZ) to downregulate DGKZ expression. The efficient silencing of DGKZ expression was verified using Immunofluorescence assay, RT-qPCR and western blot analysis (Figure 2a–d). Compared with the negative control (sh-NC), DGKZ protein expression was significantly decreased in SiHa and HeLa cells following introduction of sh-DGKZ. The proliferation of SiHa and HeLa cells was monitored by MTT method. Compared to sh-NC-transfected cells, proliferation of SiHa and HeLa cells transfected with sh-DGKZ were remarkably reduced (Figure 3a). In addition, silence of DGKZ accumulated SiHa and HeLa cells at G0/G1 phase and thus caused a significant inhibition of cell cycle progression (Figure 3b, c).

### Knockdown of DGKZ boosts the apoptosis of SiHa and HeLa cells

**Figure 4.**
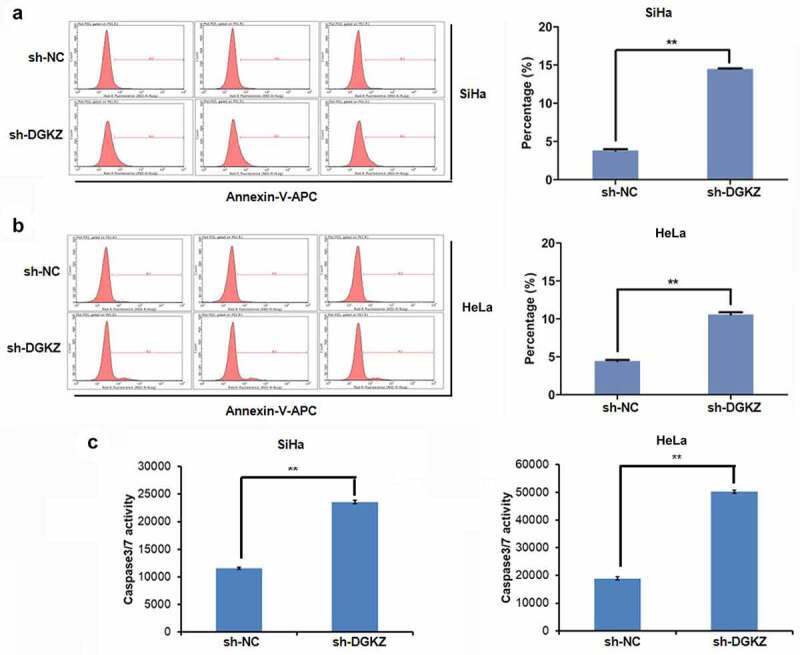
Silence of DGKZ facilitated apoptosis of SiHa and HeLa cells . (a, b) Apoptosis of SiHa or HeLa cells transfected with sh-NC or sh-DGKZ was assessed by flow cytometry via Annexin V-APC staining (**p < 0.01). (c) cCaspase3/7 activity in SiHa or HeLa cells transfected with sh-NC or sh-DGKZ was examined using Caspase-Glo 3/7 assay. (**p < 0.01)

Based on the results above, we speculated that the cell growth inhibition caused by DGKZ knockdown was partially owing to promoting cell apoptosis. To identify this hypothesis, the apoptosis of transfected SiHa and HeLa cells was analyzed by fluorescence activated cell sorting (FACS) analysis using Annexin-V-APC staining. In comparison with sh-NC transfection, it was observed that sh-DGKZ transfection led to the higher apoptosis of SiHa and HeLa cells (to SiHa: sh-NC vs sh-DGKZ: mean percentage of apoptosis cells = 3.83% vs 14.50%, P < 0.01; to HeLa: sh-NC vs sh-DGKZ: mean percentage of apoptosis cells = 4.45% vs 10.60%, P < 0.01) (Figure 4a, b).Furthermore, caspase3/7 activity directly related to cell apoptosis degree was examined using Caspase-Glo 3/7 assay. Significantly elevated caspase3/7 activity caused by DGKZ silencing was viewed in SiHa and HeLa cells (P < 0.01) (Figure 4c) .

### DGKZ accelerates cervical carcinogenesis in vivo

**Figure 5.**
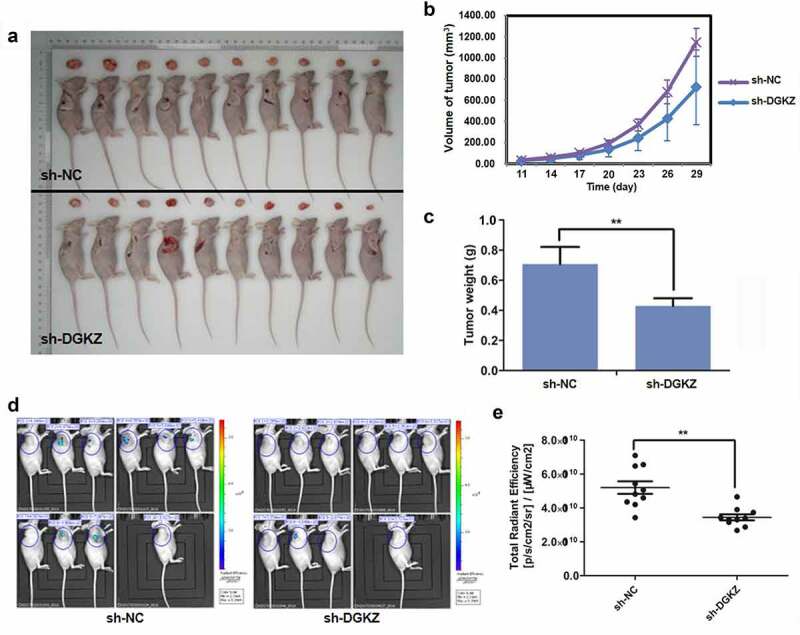
Suppression of DGKZ slowed progression of cervical cancer *in vivo*. (a) Photographs of xenografts of BALB/c nude mice for 31d post-injection. (b) Tumor volume (mm^3^) of BALB/c mice injected subcutaneously with Lentivirus-sh-DGKZ-SiHa cells or Lentivirus-sh-NC-SiHa cells. (c) Tumor weight (g) of BALB/c mice injected subcutaneously with Lentivirus-sh-DGKZ-SiHa cells or Lentivirus-sh-NC-SiHa cells. (d–e) Representative images of fluorescence imaging and analysis. (**p < 0.01)

To determine whether DGKZ functioned on tumorigenesis *in vivo*, Lentivirus-sh-DGKZ-SiHa cells or Lentivirus-sh-NC-SiHa cells were injected subcutaneously into 20 BALB/c nude mice in the subcutaneous area. BALB/c mice were killed for evaluation at 31 d after xenotransplantation (Figure 5a). In comparison with sh-NC group, DGKZ knockdown significantly decreased volumes and weights of tumors (Figure 5b, c). In order to determine xenograft tumorigenesis of BALB/c nude mice in vivo, the anesthetized BALB/c nude mice with xenotransplanted tumor were conducted to fluorescence imaging assay using small animal live imaging system (Figure 5d). The total radiant efficiency reflecting the number of xenograft tumor cells in sh-DGKZ group was conspicuously lower than that in sh-NC group (P < 0.01) (Figure 5e).

### Biological function enrichment analysis of DGKZ-related differentially expressed proteins

**Figure 6.**
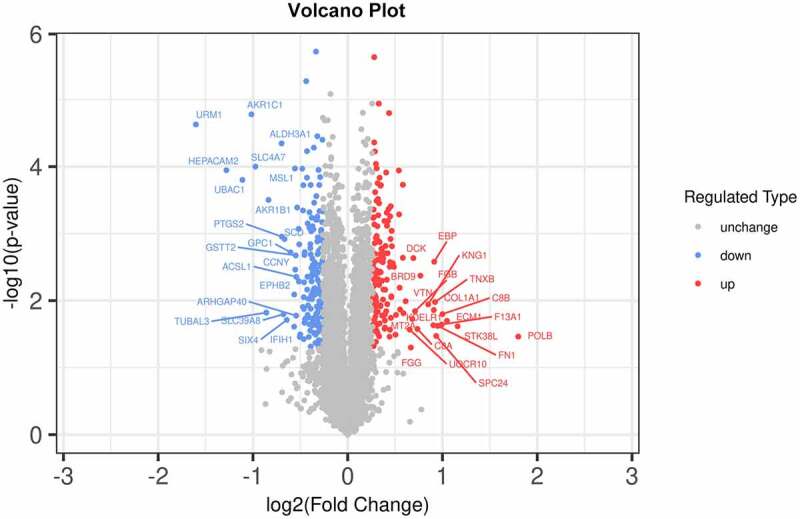
Expression profiles in sh-DGKZ-SiHa cells or sh-NC-SiHa cells. In the volcano plots, the red points represent the differentially upregulated proteins and the blue points represent the differentially downregulated proteins

To dissect the biological function of DGKZ in CC, Tandem Mass Tag (TMT)-based Quantitative Proteomics was used to investigate differentially expressed DGKZ-related proteins in Lentivirus-sh-DGKZ-SiHa cells or Lentivirus-sh-NC-SiHa cells. As demonstrated by volcano plot analysis, 314 differentially expressed proteins (DEPs) consisting of 148 up-regulated DEPs and 166 down-regulated DEPs were successfully identified (Figure 6).

**Figure 7.**
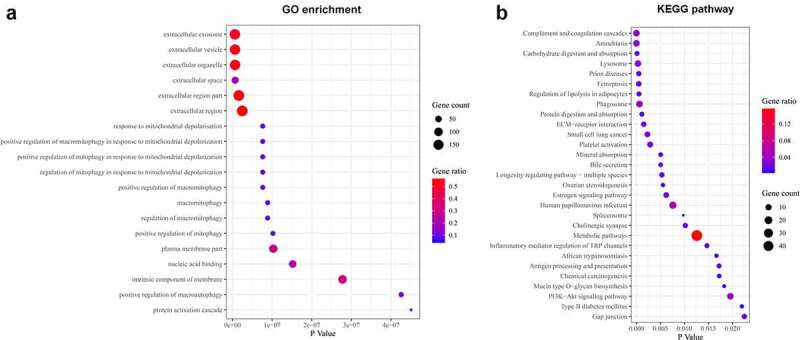
Biological processes and pathways associated with DGKZ in CC. (a) GO enrichment analysis on the different genes in cervical cancer profiles. The ordinate represents the GO items and the abscissa represents the number of different genes. (b) KEGG functional enrichment analysis of DEPs in cervical cancer expression profile. The ordinate represents the KEGG items and the abscissa represents the number of different genes

To deeply analyze the biological pathways and processes associated with DGKZ, the 314 proteins were subjected to Gene Ontology analysis and KEGG pathway enrichment. Results of GO analysis exhibited that these DEPs were markedly enriched in biological processes: macro-autophagy and macro-mitophagy (P < 0.05) (Figure 7a). Through KEGG pathway analysis, 284 pathways were identified as significantly enriched (P < 0.05), such as lysosome, phagosome and PI3K-Akt signaling pathway. The top 30 of these 284 pathways were shown using bubble diagram (Figure 7b).

### Detection and qualification of molecules involving in stress and apoptosis signaling

**Figure 8.**
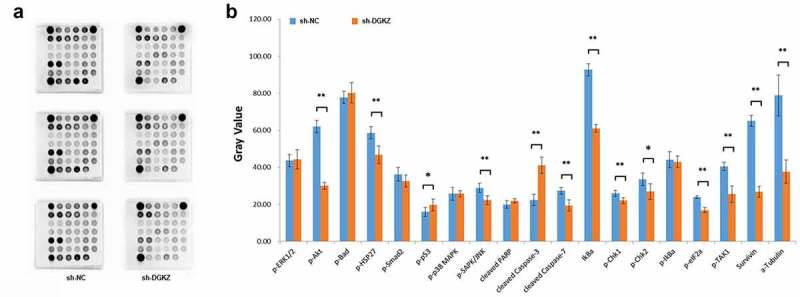
Identification of DGKZ knockdown-related gene expression. (a)The PathScan Stress and Apoptosis Signaling Antibody Array Kit was performed to assess the signaling molecules that were associated with downregulation of DGKZ in SiHa cells. (b) Quantitative array data by array analysis software. (*p < 0.05, **p < 0.01)

The stress and apoptosis signaling related molecules were analyzed using PathScan stress and apoptosis signaling antibody array kit (Figure 8a). The array allows for the simultaneous detection (in duplicate) of 19 important and relevant signaling molecules under phosphorylated or cleaved status. The data (Figure 8b) displayed that DGKZ knockdown significantly elevated the expression of p-p53, cleaved-caspase3, and suppressed the expression of p-AKT, p-HSP27, p-SAPK/JNK, cleaved-caspase7, IκBα (unphosphorylated), p-Chk1, p-Chk2, p-eIF2α, p-TAK1, Survivin and α-Tubulin in SiHa cells.

## Discussion

DGKs work as a switch for two lipid signals (DAG and PA), regulating diverse cell biological effects. The role of DGKs in lipid metabolism may be a potential mechanism in cancer progression. For example, DGKZ controls mRORC1 function to affect the colon cancer survival [11]. Downregulation of DGKZ inhibits the cell proliferation and survival in human gliomas [16]. In current investigation, we firstly discovered the potential relationship between DGKZ and CC. The high expression of DGKZ in tumor tissues from CC patients indicated the promising role of DGKZ in CC progression. Inhibition of DGKZ suppressed tumor growth, which was evidenced both *in vivo* and *in vitro*.

Early in 1998, DGKZ was reported to be regulated by protein kinase C, and may be a general control of mitogenic mechanism [17]. Cells under cell cycle progression are more sensitive to the death signal than those during quiescent stage. Even though the connection between cell cycle arrest and apoptosis has not been well investigated, cell cycle arrest always emerges along with cell apoptosis in cancer therapeutic strategy [16,18]. Caspase3 can be activated in the late G1 phase of cell cycle when cells are cyclin A/B1 negative and cyclin E positive [19]. The current investigation revealed that inhibition of DGKZ reduced cell viability and facilitated cell cycle arrest in G0/G1 phase, resulting in cell death. To further understand the role of DGKZ in CC progression, DEPs in SiHa cells with or without transfection of sh-DGKZ were enriched and analyzed by GO and KEGG biological analysis. GO analysis indicated that DEPs triggered by DGKZ depletion mostly induced macro-autophagy and macro-mitophagy, which regulated cell apoptosis and cell cycle in most cancer development [20–22]. In KEGG pathway analysis, the significant alteration was closely associated with autophagy or mitophagy including lysosome, phagosome and PI3K-Akt signaling pathway.

PathScan stress and apoptosis signaling antibody array kit was used to quantify the molecular pathways in stress and apoptosis after inhibition of DGKZ. AKT, a vital molecule in PI3K-AKT signaling pathway, induces cell survival by phosphorylating MDM2 that can combine with tumor suppressor p53 to induce degradation of p53 [23,24]. SAPKs (or named JNKs) induce apoptosis by phosphorylating Bcl-2 and Bcl-xL which evoke release of Cytochrome C in the mitochondria and then result in cascade reaction of caspase activation [25,26]. But, in some cases, JNK dedicated on cell proliferation such as: Suppression of JNK by JNK interacting protein (JIP) efficiently promotes nasopharynx cancer apoptosis [27]. Cleaved-caspase3 accumulation is an obvious and vital sign of activated cell apoptosis [28]. Nonetheless, the expression of cleaved-caspase7 was reduced in sh-DGKZ-transfected cells that and the low levels of activated caspase7 are associated with cancer poor prognosis [29]. The exact effects of caspase7 in CC still require in-depth exploration. Current data presented that downregulation of DGKZ enhanced the apoptosis and caspase3 activity in CC. IkBα (phosphorylated) initiates cell apoptosis process by accumulating NF-κB p65 transformation into cell nucleus and suppressing inhibitor of apoptosis expression [30]. IkBα (unphosphorylated) degradation provokes the activation of NF-κB pathway. Chk-1/-2 are known as indispensable protein kinases involved in cell cycle regulation and coordination DNA synthesis process [31–33]. Downregulation of Chk-α reduces proliferation of breast cancer cells and tumors [34]. eIF2α, phosphorylated by PERK, promotes cell apoptosis by inducing ATF4 activation and then upregulating CHOP expression [35]. The reduction of p-eIF2α can induce inhibition of cell apoptosis initial signaling triggered by endoplasmic reticulum disorder, and has negative effect on cell apoptosis. In the present investigation, enhancement of cell apoptosis caused by knockdown of DGKZ in CC hinted that activation of caspase3 holds the main position. TAK1 (TGF-β-activated kinase 1) induces IΚΚβ phosphorylation and activates NF-κB pathway to promote tumorigenesis [36]. Survivin, specifically expressed in tumor and embryo tissues, directly combines with caspase3/7 to inhibit its activation and thus blocks cell apoptosis [37,38]. According to current data and analysis, we supposed that PI3K-Akt and TAK1-NF-κB signaling pathways may be the potential molecular mechanisms participating the effects of DGKZ in proliferation and apoptosis in CC.

## Conclusions

To sum up, the present work identified that DGKZ worked as an oncogene for CC and preliminarily predicted the potential molecular mechanisms by which DGKZ functioned in CC. In spite of above achievement, the current work lacks more reliable studies that can confirm the underlying mechanism of DGKZ in facilitating CC progression. More importantly, in-depth study should be conducted to investigate the relationship between DGKZ expression and remission/survival of CC patients in the future, aiming to excavating the clinical values of DGKZ.

## Data Availability

All datasets used and/or analyzed during the current study are available from the corresponding author on reasonable request.
